# Ghrelin increases intake of rewarding food in rodents

**DOI:** 10.1111/j.1369-1600.2010.00216.x

**Published:** 2010-07

**Authors:** Emil Egecioglu, Elisabet Jerlhag, Nicolas Salomé, Karolina P Skibicka, David Haage, Mohammad Bohlooly-Y, Daniel Andersson, Mikael Bjursell, Daniel Perrissoud, Jörgen A Engel, Suzanne L Dickson

**Affiliations:** 1 Department of Physiology/Endocrinology, Institute of Neuroscience and Physiology, The Sahlgrenska Academy at the University of GothenburgSweden; 2 Department of Pharmacology, Institute of Neuroscience and Physiology, The Sahlgrenska Academy at the University of GothenburgSweden; 3 Department of Integrative Medical Biology, Section for Physiology, Umeå UniversitySweden; 4 AstraZeneca R&D MölndalSweden; 5 ÆternaZentarisFrankfurt, Germany

**Keywords:** Dopamine, food anticipation, motivation, obesity, reward, VTA

## Abstract

We investigated whether ghrelin action at the level of the ventral tegmental area (VTA), a key node in the mesolimbic reward system, is important for the rewarding and motivational aspects of the consumption of rewarding/palatable food. Mice with a disrupted gene encoding the ghrelin receptor (GHS-R1A) and rats treated peripherally with a GHS-R1A antagonist both show suppressed intake of rewarding food in a free choice (chow/rewarding food) paradigm. Moreover, accumbal dopamine release induced by rewarding food was absent in GHS-R1A knockout mice. Acute bilateral intra-VTA administration of ghrelin increased 1-hour consumption of rewarding food but not standard chow. In comparison with sham rats, VTA-lesioned rats had normal intracerebroventricular ghrelin-induced chow intake, although both intake of and time spent exploring rewarding food was decreased. Finally, the ability of rewarding food to condition a place preference was suppressed by the GHS-R1A antagonist in rats. Our data support the hypothesis that central ghrelin signaling at the level of the VTA is important for the incentive value of rewarding food.

## INTRODUCTION

Ghrelin, a gastric-derived peptide (Kojima *et al*. 1999), increases food intake (Wren *et al*. 2000) and has pro-obesity effects (Tschöp, Smiley & Heiman 2000). Indeed, the preprandial rise in circulating ghrelin levels in human subjects that correlates with hunger scores has been used to suggest a role in hunger and meal initiation (Cummings *et al*. 2001). These effects are exerted, at least in part, at the level of the hypothalamus (Tschöp *et al*. 2000; Cowley *et al*. 2003), especially the arcuate nucleus where the ghrelin receptor, growth hormone secretagogue receptor 1A (GHS-R1A), is expressed in abundance (Howard *et al*. 1996; Guan *et al*. 1997). GHS-R1A is also present in tegmental areas implicated in food reward and addiction, the ventral tegmental area (VTA) and the laterodorsal tegmental area (Guan *et al*. 1997). Indeed, recently we demonstrated that ghrelin injection intracerebroventricular (i.c.v.) or into the VTA or laterodorsal tegmental area stimulates parameters associated with reward-seeking behavior (Jerlhag *et al*. 2006, 2007, 2008), a finding confirmed and extended by others (Abizaid *et al*. 2006). In addition, human functional imaging studies have recently shown that peripheral ghrelin administration modulates brain responses to food images in several areas associated with reward (Malik *et al*. 2008).

The mesolimbic dopamine projections, originating from neuronal cell populations in the VTA and terminating in the ventral striatum and the prefrontal cortex, are linked to anticipatory, appetitive or approach phases of motivated behavior and are important for anticipatory food reward and food-seeking behavior (Richardson & Gratton 1998; Bassareo & Di Chiara 1999). Activation of these dopamine projections is also elicited by ingestion of rewarding/palatable foods as well as by other rewards, both natural (e.g. sex) and artificial (e.g. drugs of abuse) (Berridge & Robinson 1998). Furthermore, feeding behavior and food-reinforced responses can be disrupted by pharmacologic interference with the dopamine system (Barzaghi *et al*. 1973).

Given the role for the mesolimbic dopamine system in incentive processes related to natural rewards such as rewarding food, and also the emerging evidence that this system is a target for ghrelin, we set out to demonstrate that ghrelin increases food reward and conversely, that food reward is suppressed in genetic and pharmacologic models of suppressed ghrelin signaling. Thus, we investigated whether (1) intra-VTA ghrelin injection increases the intake of rewarding food and/or normal chow; (2) GHS-R1A knockout (KO) mice and GHS-R1A antagonist-treated rats show reduced preference for rewarding food; (3) accumbal dopamine release induced by rewarding food is suppressed in GHS-R1A KO mice; (4) VTA lesion disrupts food intake and the motivation to eat palatable food following ghrelin treatment; and (5) the ability of rewarding food to condition a place preference is suppressed by GHS-R1A antagonists.

## MATERIALS AND METHODS

### Animals

Adult male NMRI mice (25–35 g BWt, B & K, Sollentuna, Sweden) were used for locomotor activity and feeding experiments following intra-VTA injections of ghrelin. Female GHS-R1A KO and wild-type littermate mice (20–25 g BWt, generation of GHS-R1A KO: see supplementary Fig. S1; details provided at the end) were used for food preference experiments. For microdialysis experiments, male GHS-R1A KO and wild-type littermate mice were used.

Adult (200–220 g BWt) Sprague-Dawley rats (B & K) were used for food preference and conditioned place preference (CPP) studies. Standard chow (Harlan Teklad; Norfolk, England) and water were available *ad libitum* unless otherwise stated. The animal room was maintained on a 12/12 hour light/dark cycle, at 20°C and 50% humidity. The local Ethics Committee for Animal Experiments (Gothenburg, Sweden) approved all procedures.

### Drugs

Acetylated rat ghrelin (Bionuclear, Bromma, Sweden) was dissolved in vehicle solution (Ringer, Merck KGaA, Darmstadt, Germany) and administered bilaterally into the VTA at a dose of 2 µg/mouse. This dose has been shown to activate the mesolimbic dopamine system (Jerlhag *et al*. 2006). Acetylated human ghrelin (gift from Rose Pharma A/S, Copenhagen, Denmark) dissolved in saline was administered as a single i.c.v. injection (1 µg) in the VTA lesion studies in rats. The dose of JMV2959 (ÆternaZentaris), a GHS-R1A antagonist (Moulin *et al*. 2007), was dissolved in saline and administered i.p. to rats at a dose of 12 mg/kg per day in the food preference experiment and at a dose of 1 mg/kg in the CPP experiment.

### Surgery

NMRI mice were implanted with bilateral guide cannulae into the VTA and rats into the lateral ventricle using steriotaxic co-ordinates as described previously (Jerlhag *et al*. 2007; Salomé*et al*. 2009). In rats coordinates used for VTA lesion relative to bregma were: 6.0 mm posterior, ±0.6 mm lateral and 8.4 mm below the surface of the brain (Paxinos & Watson 1986). Bilateral lesions involved ibotenic acid injection (0.06 M in saline, 200 nl, Sigma). Sham animals received saline only. Wild-type littermate controls and GHS-R1A KO mice were implanted with a unilateral microdialysis probe (Waters *et al*. 1993) positioned in the N.Acc. for measurement of extracellular dopamine levels as described previously (Jerlhag *et al*. 2006). All animals were individually housed following implantation of injection cannulae or microdialysis probes.

### Food intake/preference measurements in genetic and pharmacologic models of suppressed ghrelin signaling

Individually housed wild-type and GHS-R1A KO mice were put on a free choice *ad libitum* feeding paradigm, consisting of chow and peanut butter for 7 days. Individually housed rats were given a free choice of *ad libitum* chow and Ensure® chocolate drink for 10 days. This palatable drink induces obesity in rats (Levin & Dunn-Meynell 2002). On day 4, rats received i.p. injections of JMV2959 or saline. Animals that did not spontaneously consume Ensure® were excluded (cut-off at 10% total caloric intake).

### Accumbal dopamine release following acute presentation of palatable food

The microdialysis technique enables concentration measurements of neurotransmitters in awake, freely moving animals. Two days following surgery, wild-type and GHS-R1A KO mice were connected to a microperfusion pump (U-864 Syringe Pump, AgnThós AB, Lidingö, Sweden) and perfused with Ringer solution at a rate of 1.5 µl/min. After 1 hour of habituation to the microdialysis perfusion set-up, perfusion samples were collected every 20 minutes.

Four samples were collected prior to peanut butter exposure and a further four samples were taken after exposure. The average baseline was calculated from the last three samples prior to peanut butter exposure. All of the animals were naïve to the peanut butter and had been allowed to consume only 1 g of chow during the dark period prior to the dialysis experiment. The exposed tip of the dialysis membrane (20 000 kDa cut off with an o.d./i.d. of 310/220 µm, HOSPAL, Gambro, Lund, Sweden) of the probe was 1 mm. The dopamine levels in the dialysates were determined by high performance liquid chromatography with electrochemical detection. A pump (Gyncotec P580A; Kovalent AB; V. Frölunda, Sweden), an ion exchange column (2.0 × 100 mm, Prodigy 3 µm SA; Skandinaviska GeneTec AB; Kungsbacka, Sweden) and a detector (Antec Decade; Antec Leyden; Zoeterwoude, the Netherlands) equipped with a VT-03 flow cell (Antec Leyden) were used. The mobile phase (pH 5.6), consisting of sulfonic acid 10 mM, citric acid 200 mM, sodium citrate 200 mM, 10% EDTA, 30% MeOH, was vacuum filtered using a 0.2 µm membrane filter (GH Polypro; PALL Gelman Laboratory; Lund, Sweden). The mobile phase was delivered at a flow rate of 0.2 ml/min passing a degasser (Kovalent AB), and the analyte was oxidized at +0.4 V.

Following the microdialysis experiment, the mice were decapitated and the brains were sectioned (50 µm thickness) and the location of the probe was determined by microscopic observation. Only mice with probe placement in the N.Acc. Shell were included in the statistical analysis.

### Conditioned place preference for palatable food

The CPP test was performed in satiated rats (*n* = 18) using an apparatus comprised of two compartments with distinct visual and tactile cues and illuminated by 40 lux. On day 1 (pre-test), the animals were free to explore the entire apparatus for 10 minutes and initial preference was scored. During the conditioning phase (day 2–6, 8, 10, 19 and 20) animals were confined for 20 minutes to one of the two compartments in the morning and to the other compartment in the afternoon (18 sessions total). For place conditioning, the least preferred compartment (determined from the pre-test) was always paired with 5 g of rewarding food (Ms, Marabou, Kraft Foods, Upplands Väsby, Sweden), and the other side was paired to standard chow. All rats consumed the chocolate pellets during the conditioning sessions, and rarely consumed chow. The conditioning phase was balanced so that the conditioned stimulus was alternated between morning and afternoon sessions. On day 22, rats were injected (i.p.) with vehicle (saline) or with JMV2959 (1 mg/kg) 10 minutes before being placed in the CPP apparatus for 10 minutes. All rats were habituated for 4 days to the rewarding food prior to the pre-test and to i.p. injection on at least six occasions prior to the test day. The behavior of the animals was recorded with a digital camera (Canon MV900) and time spent in each compartment was determined by visual analysis of the video.

### Acute food intake/preference measurements following intra-VTA injections of ghrelin to mice

To investigate the acute effects of ghrelin within the VTA on the intake and preference for palatable food, ghrelin (2 µg total) was administered bilaterally into the VTA to the NMRI mice. Locomotor (120 minutes) activity and chow or peanut butter consumption (60 minutes) was registered in eight sound attenuated, ventilated and dimly lit locomotor boxes (420 × 420 × 200 mm, Kungsbacka mät- och reglerteknik AB, Fjärås, Sweden) as previously described (Jerlhag *et al*. 2006). Locomotor activity was defined as the accumulated number of new photocell beams interrupted during a 60-minute period. On the day of the experiment, the mice were allowed to habituate to the environment in the box for 60 minutes before ghrelin/vehicle challenge and exposure to chow or peanut butter. To reduce the influence of injection-induced hyper-motility, the registration of locomotor activity started 5 minutes after the last ghrelin/vehicle administration. The mice were not naïve to peanut butter as they had been given free access for 1 hour everyday for 5 days prior to the study.

### Measurements of food intake and food exploration following i.c.v. ghrelin injection in VTA-lesioned rats

Food consumption and body weight gain were monitored for 7 days following surgery in sham- and VTA-lesioned rats. On day 8 after surgery, rats were administered ghrelin (1 µg; i.c.v.) or vehicle and 4 hours chow consumption was measured. On days 13–15 post-surgery, the rats were administered the same treatment and were placed in an open field chamber containing an open eppendorf tube filled with peanut butter. Exploration time (defined by eating or active pursuit of peanut butter, analyzed from video recordings) and peanut butter intake over a 10 minutes period were measured. Prior to surgery, the rats were familiarized with the eppendorf tube and the palatable food for 5 days.

### Statistics

Data were analyzed using Student's *t*-tests, repeated-measures two-way analysis of variance (ANOVA) or two-way ANOVA followed by Bonferroni *post hoc* test. The significance level was *P* < 0.05 for all experiments. All data are presented as means ± standard error of the mean.

## RESULTS

**Figure 1:**
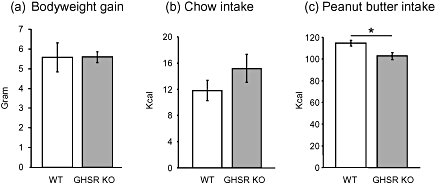
(a) Body weight gain; (b) chow consumption; and (c) peanut butter consumption in growth hormone secretagogue receptor 1A knockout (GHS-R1A KO) and wild-type mice offered a free choice between *ad libitum* chow and peanut butter. *n* = 8 (wild-type) and *n* = 5 (GHS-R1A KO), *P < 0.05, Student's *t*-test

**Figure 2:**
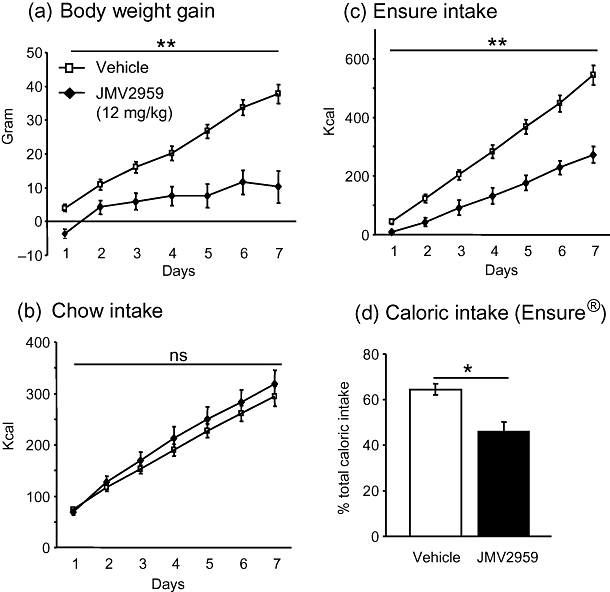
Effects of daily peripheral treatment with a selective growth hormone secretagogue receptor 1A (GHS-R1A) antagonist (JMV2959) (12 mg/kg) to rats on (a) body weight gain; (b) cumulative chow intake; (c) intake of Ensure®; and (d) preference for Ensure®. *n* = 5–6 per group, a–c **P < 0.01, repeated-measures two-way analysis of variance, d *P < 0.05, Student's *t*-test

When offered a free choice diet (chow/peanut butter) for 7 days, GHS-R1A KO mice consumed 10% less peanut butter compared with wild-type littermate mice (*P* < 0.05, Fig. 1c); chow intake and body weight did not differ (Fig. 1a,b). In a similar free-choice paradigm (chow/Ensure® for 10 days), JMV2959-treated rats consumed 50% less Ensure® and gained considerably less body weight than vehicle-treated controls (both *P* < 0.01, Fig. 2a,c). Chow intake was unaffected by JMV2959 treatment (Fig. 2b). Total 7-day caloric intake (chow plus Ensure®) was decreased in JMV2959-treated rats compared with vehicle-treated rats (591 ± 28 kcal and 839 ± 20 kcal, respectively, *P* < 0.001, Student's *t*-test). Thus, vehicle-treated rats consumed 65 ± 3% of their total caloric intake from Ensure®; whereas, JMV2959-treated rats consumed considerably less of their total caloric intake (46 ± 4%) from Ensure® (*P* < 0.05, Fig. 2d). Food efficiency was lower in JMV2959-treated compared with vehicle-treated rats (veh: 0.045 ± 0.003 and JMV2959: 0.015 ± 0.008, *P* < 0.05, Student's *t*-test).

In wild-type mice (*n* = 6) peanut butter increased the accumbal dopamine levels and this effect was not observed in GHS-R1A KO (*n* = 7) mice (treatment F(1,11) = 4.91, *P* < 0.05; time F(6,66) = 0.60, *P* = 0.733; treatment-time interaction F(6,66) = 2.092, *P* = 0.066). This difference was largely reflected by the difference at the 40 minutes time point following peanut butter presentation (wild-type: 143 ± 22 % from baseline, GHS-R1A KO: 80 ± 13% from baseline, *P* < 0.05 Student's *t*-test). Peanut butter consumption over the 80 minutes period was, however, not different between wild-type and GHS-R1A KO mice (data not shown).

**Figure 3:**
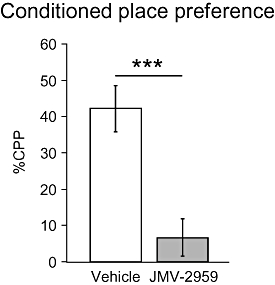
Effects of peripheral pre-treatment (10 minutes prior to test) with a ghrelin antagonist (JMV2959, 1 mg/kg) on the ability of rewarding food to condition a place preference. The increased preference [% conditioned place preference (CPP)] was calculated using the following formula: ((test − pre-test)/(total time − pre-test)) × 100. *n* = 9 in each group, ***P < 0.001, Student's *t*-test

The chocolate pellets induced a CPP response in vehicle- but not in JMV2959-treated rats (*P* < 0.001, Fig. 3). The time spent in the rewarding food-paired compartment was not different between vehicle- and JMV2959-treated rats in the pre-test (Veh: 224 ± 22 second, JMV2959: 262 ± 19 second) and there was no initial preference for one particular compartment in the pre-test (51.7 ± 4.2% versus 48.2 ± 4.2%).

**Figure 4:**
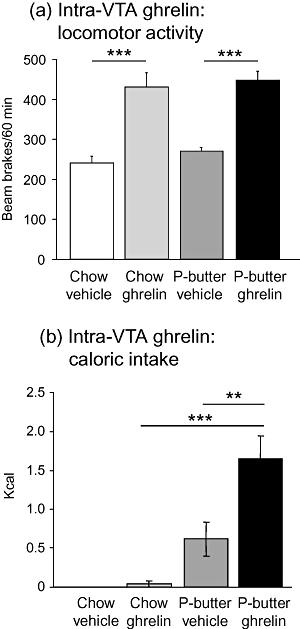
Effects of intra-ventral tegmental area (VTA) injections of ghrelin or vehicle on 60 minutes locomotor activity and feeding in mice exposed to different kinds of foods. Bilateral intra-VTA administration of ghrelin increased both (a) locomotor activity in the presence of both chow and peanut butter and (b) the intake of peanut butter but not chow compared with vehicle administration. *n* = 8 for all groups (a) ***P < 0.001, effect of treatment two-way analysis of variance (ANOVA) (b) **P < 0.01, ***P < 0.001, two-way ANOVA followed by Bonferroni *post-hoc* test

Intra-VTA administration of ghrelin to NMRI mice increased 60 minutes locomotor activity in the presence of either standard chow or peanut butter (both *P* < 0.001, Fig. 4a). However, intra-VTA ghrelin administration increased caloric intake relative to vehicle treatment only in mice fed with peanut butter and not in mice fed with standard chow (*P* < 0.001, Fig. 4b).

**Figure 5:**
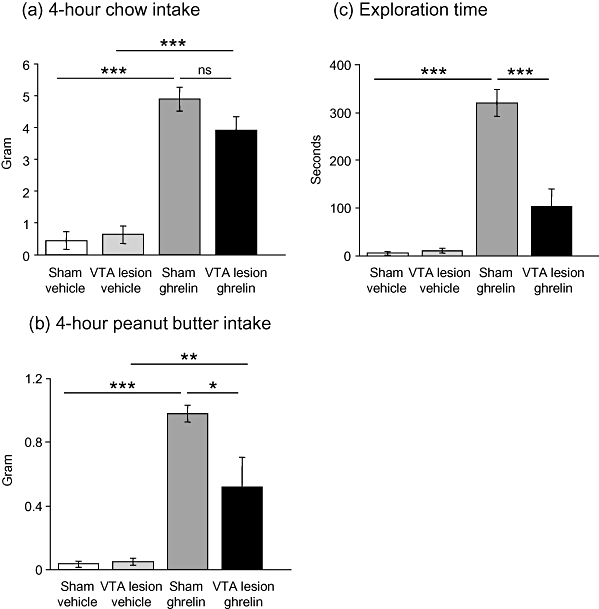
Effects of bilateral ventral tegmental area (VTA) lesion on ghrelin-induced feeding and food exploratory activity. (a) I.c.v. ghrelin treatment increased 4-hour chow intake in both sham and VTA-lesioned rats compared with vehicle treatment. Ghrelin-induced chow consumption did not differ between sham and VTA-lesioned rats. (b) I.c.v. ghrelin injection increased the consumption of peanut butter contained in an eppendorf tube in both sham and VTA-lesioned rats compared with vehicle treatment. The consumption of peanut butter following ghrelin injection was, however, attenuated in VTA-lesioned rats compared with sham rats as was (c) the time spent exploring the peanut butter/eppendorf setup. *P < 0.05, **P < 0.01, ***P < 0.001, two-way analysis of variance followed by Bonferroni *post-hoc* test

Chemical lesion of the VTA in rats did not affect body weight gain (sham: 55 ± 3 g; lesioned: 53 ± 3 g) or consumption of chow (sham: 140 ± 4 g; lesioned: 138 ± 5 g) measured over the 7 days following surgery indicating that the lesion did not induce hypophagia *per se*. Moreover, no difference in baseline locomotor activity was found between sham and VTA-lesioned rats (702 ± 83 and 717 ± 136 beam brakes/120 minutes, respectively). I.c.v. ghrelin injection increased 4-hour standard chow intake in both sham and VTA-lesioned rats compared with vehicle treatment (both *P* < 0.001, Fig. 5a). However, i.c.v. ghrelin-induced chow intake did not differ between sham and VTA-lesioned rats (Fig. 5a). I.c.v. ghrelin injection increased the consumption of peanut butter (contained in an eppendorf tube) in both sham and VTA-lesioned rats compared with vehicle treatment (*P* < 0.001 and *P* < 0.01, respectively, Fig. 5b). Ghrelin-induced peanut butter consumption was, however, attenuated in VTA-lesioned rats compared with sham rats (*P* < 0.05, Fig. 5b). The time spent exploring the peanut butter/eppendorf setup was considerably decreased in ghrelin-treated VTA-lesioned rats compared with ghrelin-treated sham rats (*P* < 0.001, Fig. 5c). The increased exploration time was not coupled to actual eating but rather to the effort of trying to eat and access remaining peanut butter left at the bottom of the eppendorf tube. When individuals that did not explore/consume any of the peanut butter were excluded, no difference in consumption of peanut butter could be found between ghrelin-treated sham and VTA-lesioned rats (sham ghrelin: 0.98 ± 0.05 g; lesioned ghrelin: 0.78 ± 0.15 g, *P* = 0.2, Student's *t*-test). Importantly, the time spent exploring the peanut-filled eppendorf (including eating) was still decreased by 52% in VTA-lesioned rats compared with sham rats (sham ghrelin; 321 ± 28 s, lesioned ghrelin; 154 ± 27 s, *P* < 0.01, Student's *t*-test). The proportion of rats not interested in peanut butter following ghrelin administration was greater (2/6) in the VTA-lesioned group than in the sham group (0/5).

## DISCUSSION

In the present study genetic, pharmacologic and surgical rodent models of altered ghrelin signaling were used to provide evidence that ghrelin action at the level of the VTA, a key node in the mesolimbic reward circuitry, is important for the intake of and motivation to obtain palatable/rewarding food. When allowed a free choice between rewarding/palatable food and standard chow, both rats and mice spontaneously consumed a greater proportion of their calories from the rewarding food. However, genetic deletion of GHS-R1A in mice (GHS-R1A KO) or treatment with a GHS-R1A antagonist in rats for 7 days suppressed the intake of rewarding food without influencing chow intake. Given that the intake of rewarding food is driven not only by the need to balance energy expenditure but also by its rewarding properties, an obvious interpretation of these results is that ghrelin acts not only at the level of the hypothalamus but also via the reward systems of the brain to increase the consumption of rewarding foods. Consistent with this hypothesis, accumbal dopamine release, induced by rewarding food, was absent in GHS-R1A KO mice. Moreover, direct injection of ghrelin into the VTA of mice increased the consumption of rewarding food without impacting on chow intake. By contrast, VTA-lesioned rats displayed reduced intake of rewarding food that was accompanied by less explorative behavior of the food following ghrelin treatment compared with sham rats. Finally, we performed a CPP study in which rats learn to associate reward from food with a given environment. We found that the ability of rewarding food to condition a place preference is greatly suppressed in rats treated with a GHS-R1A antagonist.

Although GHS-R1A KO mice consumed 10% less rewarding food (kcal peanut butter) than wild-type mice, this did not result in a concomitant decrease in body weight during this 7-day test period. Clearly, 7-day exposure to rewarding food was insufficient to induce a difference in weight gain, as predicted from previous studies in which it took several weeks for these mice to show resistance to diet-induced obesity (Wortley *et al*. 2004). In contrast to the genetic studies, pharmacologic suppression of ghrelin signaling in adult rats, using a GHS-R1A antagonist JMV2959, not only suppressed intake of rewarding food (Ensure®) in a free choice (chow/Ensure®) paradigm, but also caused a suppression of body weight. The discrepancy in body weight between the genetic and pharmacologic studies most likely reflects the development of compensatory mechanisms in the GHS-R1A KO mice, mechanisms that are not in operation when the central ghrelin signaling system is acutely suppressed (over 1 week) by the GHS-R1A antagonist. This would imply that the GHS-R1A antagonist is able to override the homeostatic mechanisms controlling the energy balance, possibly involving suppressed endogenous ghrelin action at the level of the mesolimbic reward system.

In support of a mesolimbic site of action of ghrelin for increasing the intake of rewarding food, we found that intra-VTA ghrelin injection to mice increased the intake of rewarding food, but not chow, during the 60 minutes period after injection. Indeed, ghrelin injection to these sites increases accumbal dopamine release and locomotor activity, indicating that ghrelin activates the mesolimbic dopamine system (Jerlhag *et al*. 2006, 2007, 2008). We confirmed the locomotor stimulatory activity following intra-VTA ghrelin administration both in chow- and peanut butter-fed mice, providing a positive control for ghrelin's biological effects at this dose and via this route. Our hypothesis that ghrelin acts at the level of the mesolimbic reward system to influence food reward is further supported by studies showing that GHS-R1A KO mice do not display an accumbal dopamine response following presentation of palatable food. Indeed, the mesolimbic dopamine system is likely to be one of the areas showing ghrelin-induced changes in activity following presentation of visual food cues in human functional imaging studies (Malik *et al*. 2008). In contrast to the study by Naleid *et al*. (2005) in rats, we did not detect an increase in chow intake 1 hour following ghrelin administration into the VTA of mice. This may reflect differences in the feeding setup used and/or species differences as our studies were performed in mice in a novel environment (the locomotor activity boxes). Thus, whereas i.c.v. injection of ghrelin to rodents is able to increase intake of chow (this study; Tschöp *et al*. 2000; Wren *et al*. 2000), qualities of the food that reflect its palatable and rewarding properties appear to be important for determining intake when ghrelin is administered directly into the VTA.

Further evidence that central ghrelin signaling at the level of the mesolimbic reward system is important for the intake of rewarding food was sought by administering ghrelin to rats with VTA lesion. Although a rather crude approach, we found that the volumes and doses of excitotoxin used for the VTA-lesion did not affect spontaneous feeding or body weight in comparison with sham rats and that i.c.v. ghrelin-induced feeding was only suppressed in rats exposed to rewarding food (and not standard chow). In this experiment, the rewarding food was contained inside an open eppendorf tube such that exploration time could be assessed as a measure of motivation to eat. We found that VTA-lesioned rats did not differ from sham rats in the time spent exploring the eppendorf containing peanut butter following vehicle injection, but that ghrelin-induced exploratory time was greatly suppressed. The locomotor activity of the VTA-lesioned rats was found to be identical to that of the sham rats at baseline indicating that the suppressed feeding response in the VTA-lesioned rats was not a consequence of a general suppression of locomotor activity. Collectively, these studies suggest that ghrelin action at the level of the VTA impacts on food intake by influencing the motivation to eat rewarding foods.

The CPP test is commonly used to demonstrate reward, especially from addictive drugs, but has also been used to demonstrate reward associated with rewarding foods (Herzig *et al*. 2005). Just as shown previously for alcohol (Jerlhag *et al*. 2009), the ability of rewarding foods to condition a place preference was abolished in rats treated peripherally with a GHS-R1A antagonist. Consistent with suppressed reward, accumbal dopamine release induced by both alcohol (Jerlhag *et al*. 2009) and rewarding food (present article) are both absent in GHS-R1A KO mice. Collectively, these data suggest that reward from alcohol and food are both dependent upon the central ghrelin signaling system.

In the present article, we focused on the rewarding properties of food that are dependent on GHS-R1A signaling. It will be interesting to discover whether the effects of ghrelin on food reward could also be influenced by desacyl ghrelin, a biologically active form of ghrelin that has modest orexigenic effects (when administered centrally), that are independent of GHS-R1A (Toshinai *et al*. 2006). In this context, it will also be interesting to discover whether food reward is altered in physiological states in which the activity of enzymes such as ghrelin-o-actyl transferase (GOAT) are altered. GOAT increases the acyl-/desacyl–ghrelin ratio in plasma (Yang *et al*. 2008), although it remains to be determined whether it is expressed centrally and whether its activity in brain is important for food reward or other aspects of food intake.

In summary, we used a number of complementary approaches to demonstrate the importance of the central ghrelin signaling system at the level of the VTA for increasing the intake of rewarding food. Taken together with our recent observation that the central ghrelin signaling system is required for drug reward (Jerlhag *et al*. 2009), the emerging hypothesis is that this system may be important for increasing the incentive value of natural (e.g. food) and artificial rewards (e.g. alcohol). Our demonstration that the intake of rewarding food can be suppressed by ghrelin antagonists suggests that suppressed ghrelin signaling at the level of the reward system may have therapeutic benefit for suppressing the intake of rewarding food.

## SUPPORTING INFORMATION

**Figure S1** Schematic diagram of the disrupted region containing the GHS-R1A gene and the targeting vector.
