# Supplementary material for: Ghrelin increases intake of rewarding food in rodents
Source: Addict Biol. 2010 Jul;15(3):304–11. doi: 10.1111/j.1369-1600.2010.00216.x (PMC2901520; doi:10.1111/j.1369-1600.2010.00216.x)
Supplement: Supplementary file 1 [file adb0015-0304-SD1.ppt]

## Slide 1
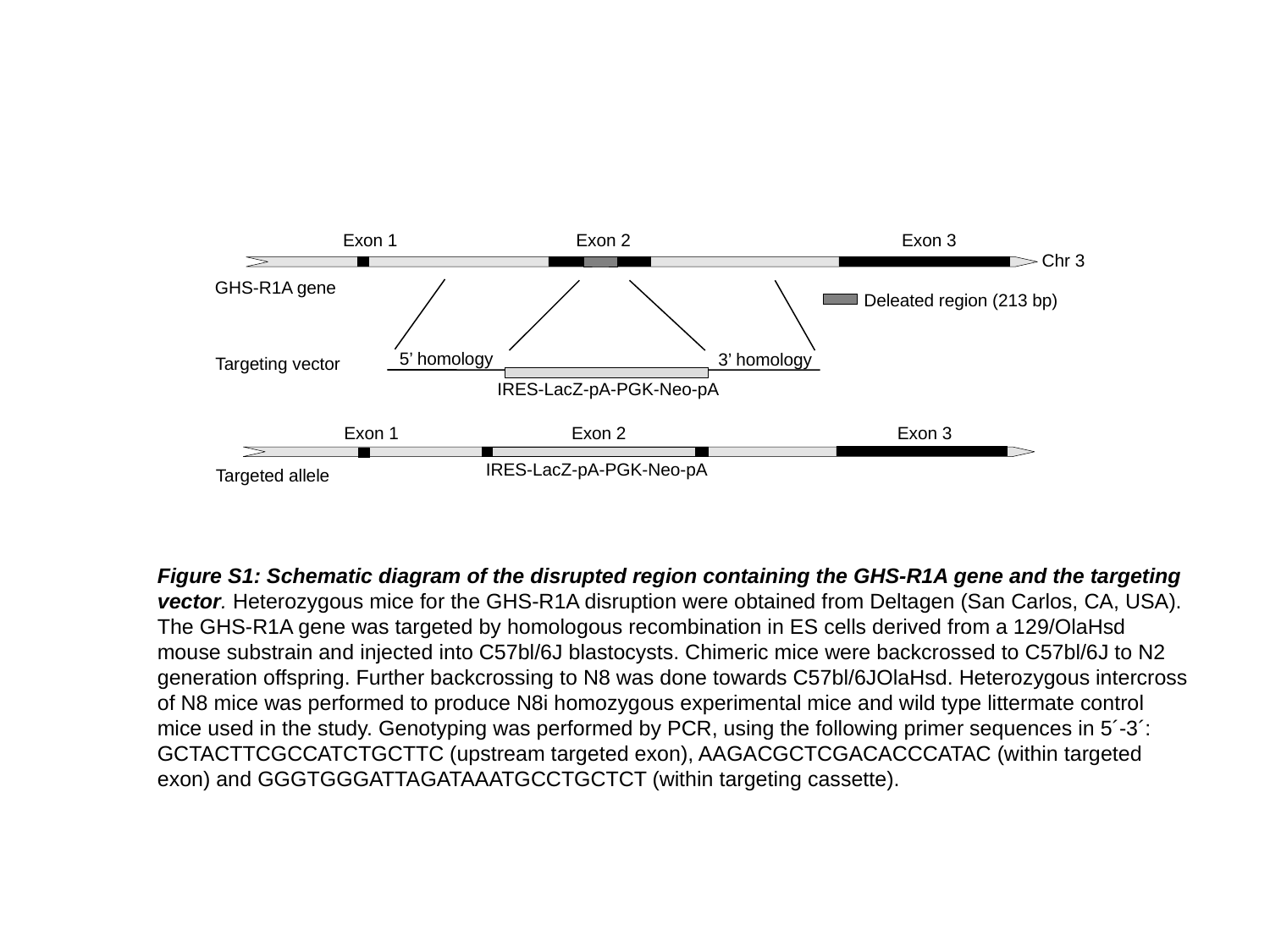

Exon 1
Exon 2
Exon 3
Chr 3
GHS-R1A gene
Deleated region (213 bp)
5’ homology
3’ homology
Targeting vector
IRES-LacZ-pA-PGK-Neo-pA
Exon 1
Exon 2
Exon 3
IRES-LacZ-pA-PGK-Neo-pA
Targeted allele
Figure S1: Schematic diagram of the disrupted region containing the GHS-R1A gene and the targeting vector. Heterozygous mice for the GHS-R1A disruption were obtained from Deltagen (San Carlos, CA, USA). The GHS-R1A gene was targeted by homologous recombination in ES cells derived from a 129/OlaHsd mouse substrain and injected into C57bl/6J blastocysts. Chimeric mice were backcrossed to C57bl/6J to N2 generation offspring. Further backcrossing to N8 was done towards C57bl/6JOlaHsd. Heterozygous intercross of N8 mice was performed to produce N8i homozygous experimental mice and wild type littermate control mice used in the study. Genotyping was performed by PCR, using the following primer sequences in 5´-3´: GCTACTTCGCCATCTGCTTC (upstream targeted exon), AAGACGCTCGACACCCATAC (within targeted exon) and GGGTGGGATTAGATAAATGCCTGCTCT (within targeting cassette).
